# Myectomy for Giant Zenker’s Diverticulum

**DOI:** 10.5152/tjg.2025.25503

**Published:** 2025-11-21

**Authors:** Bahri Abayli, Yeliz Simsek, Ali Ilker Ozer, Adnan Kuvvetli, Begum Seyda Avci, Akkan Avci

**Affiliations:** 1 Department of Gastroenterology and Hepatology, Seyhan Devlet Hospital, Adana, Türkiye; 2 Department of Emergency Medicine, Adana City Research and Training Hospital, Health Science University, Adana, Türkiye; 3 Department of General Surgery, Adana City Research and Training Hospital, Health Science University, Adana, Türkiye; 4 Department of Internal Medicine, Adana City Research and Training Hospital, Health Science University, Adana, Türkiye

Dear Editor,

Zenker’s diverticulum (ZD) is a pseudodiverticulum formed by the herniation of mucosal and submucosal structures. It occurs in the anatomically weak Killian triangle located in the transition zone between the pharynx and esophagus.^1^ Zenker’s diverticulum is most commonly diagnosed around the age of 70, but is rarely seen below the age of 40, with an estimated prevalence of 0.002 in the general population. Zenker’s diverticulum is usually classified into 3 groups based on their cranio-caudal length: small (less than 2 cm), medium (2-4 cm), and large (greater than 4 cm).^1^ The most common clinical findings are dysphagia, regurgitation, aspiration, cough, rumbling in the neck (borborygmi), choking sensation, bad breath, weight loss, and hoarseness.^1^ Although videofluoroscopy and dynamic contrast-enhanced swallowing imaging are useful diagnostic modalities, upper Gastrointestinal endoscopy is considered the essential and usually sufficient method in modern clinical practice.^1^^,^^2^ Open surgical diverticulotomy, rigid endoscopic diverticulotomy, flexible endoscopic technique, and Zenker’s peroral endoscopic myotomy (Z-POEM) are the treatment modalities. Among these, endoscopic techniques are considered the first-line approaches; however, their effectiveness and recurrence rates are still not clearly established. Reported recurrence rates were approximately 10%, particularly after Z-POEM.^2^ Data on giant ZD are insufficient and not reported separately.

We presented 3 cases of giant Zenker’s diverticulum that were successfully treated with endoscopic myectomy, with complete symptom resolution confirmed by the Kothari–Haber Scoring System. The Kothari–Haber scoring system is a simplified tool to objectively assess treatment success, with scores ranging from 0 to 16. The clinical courses are detailed below. Approval was received from the Scientific Research Ethics Committee of Adana City Training and Research Hospital (approval number: 707, date: 21.08.2025).

## Case 1

**Figure 1.**
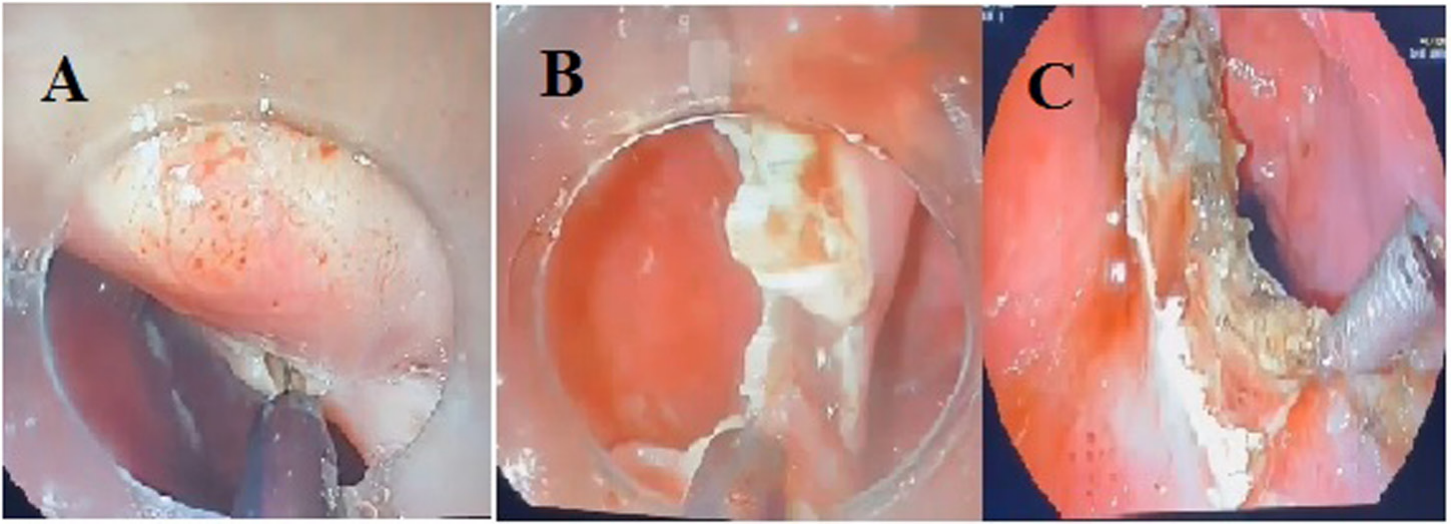
Endoscopic submucosal dissection technique. A and B: The septal muscle layer, encompassing both walls of the esophagus, was resected in a U-shaped fashion from the roof to the base of the diverticulum. The esophageal septum was carefully mobilized from the esophageal wall to the base of the esophagus using a hook blade. C: Following myectomy, prophylactic clipping was performed to support areas at risk for perforation or muscle weakness.

An 88-year-old male patient presented with symptoms of difficulty in swallowing solid and liquid foods, a cough, regurgitation, bad breath, and a 14 kg weight loss over the past year. Physical examination revealed a palpable, non-tender cervical mass with a soft consistency and no overlying skin changes. He had a history of hypertension. The patient was initially treated with Z-POEM. Two months later, his symptoms recurred. Seven months after Z-POEM, endoscopic evaluation demonstrated the presence of a giant ZD, and a myectomy was performed. An endoscopic submucosal dissection technique was used to perform the myectomy (Figure 1). In this technique, the septal muscle layer, involving both walls of the esophagus, was resected in a U-shaped manner from the roof to the base of the diverticulum. Zenker’s diverticulum septum was carefully mobilized from the esophageal wall to the base of the ZD using a hook knife. Following the myectomy, prophylactic clipping was performed to support areas deemed at risk for perforation or muscular weakness. The patient’s Kothari–Haber score was 11 before the procedure. At the 10-day follow-up, his symptoms were completely resolved, and his Kothari–Haber score was 0. One year after the myectomy, the patient gained 10 kg.

## Case 2

A 79-year-old woman presented with difficulty swallowing semi-solid foods for the last year. She also experienced a cough, regurgitation, and a 15 kg weight loss over the past year. She had a history of colon cancer surgery 28 years ago and hypertension. The patient underwent Z-POEM. She was symptom-free during the first month of follow-up period. At 1 month, her symptoms recurred, endoscopic examination demonstrated a giant ZD, and she underwent myectomy. All symptoms resolved on the fifth postoperative day. Before the procedure, the patient’s Kothari–Haber score was 8. After myectomy, we observed that the Kothari–Haber score was 0. Four months after the procedure, her symptoms completely resolved. The patient gained 12 kg.

## Case 3

A 72-year-old man presented with a 2-and-a-half-year history of difficulty swallowing both solid and liquid foods, a 20 kg weight loss in the past year, regurgitation, a cough, and bad breath. He had a history of benign prostatic hyperplasia. The patient underwent Z-POEM. After a 1-month symptom-free period, his symptoms recurred. Endoscopic examination revealed a giant ZD, and a myectomy was performed. On the fifth day after the procedure, all his symptoms resolved. Before the procedure, the patient’s Kothari–Haber score was 10. We observed that the Kothari–Haber score was 1 at the 6-month follow-up.

All 3 patients above were informed about their medical history, current findings, and disease, and their written consent was obtained. The study also obtained ethical committee approval from the Ethics Committee of University of Health Science, Adana City Research and Training Hospital (Approval No.: 707; Date: August 21, 2025).

The management of Zenker’s diverticulum is still unclear. Endoscopic approaches use laser, cautery, or stapling devices for myotomy or myectomy. The literature has rarely reported that myotomy techniques, including Z-POEM, can lead to complications such as subcutaneous emphysema, pneumomediastinum, and bleeding. Recurrence of symptoms is a significant limitation in terms of long-term effectiveness.^2^ Myectomy is a newer endoscopic approach procedure, and cases are often presented as case series in the literature. In the study of Pang et al,^3^ they performed myectomy on 20 patients and found that the treatment success rate was higher than in those who underwent myotomy. No recurrence was observed in any myectomy patients.^3^ In another study, a diverticulum larger than 6 cm was successfully treated with 2 sessions of myectomy.^4^ There were examples of giant ZD cases treated with Z-POEM in the literature.^3^^,^^5^ In our case series, 3 patients had previously undergone Z-POEM and experienced recurrences. Although data in the literature are limited, the failure of Z-POEM in giant Zenker’s diverticula may be explained by technical factors such as incomplete septotomy, difficulties in tunneling, and impaired visualization and maneuverability due to the large pouch. During the recurrence period, giant ZDs (8-10 cm) were observed in our patients. This finding suggests that Z-POEM may not be an effective treatment for giant ZDs.

We successfully utilized endoscopic myectomy for the treatment of these giant ZDs. To our knowledge, this is the first case series in the literature demonstrating the use of myectomy in the treatment of such large, recurrent ZDs. We successfully treated our patients with endoscopic myectomy and observed no complications or recurrence during the 6-month follow-up period. The relatively short follow-up period of 6 months represents a limitation of the present study. In addition, although outcomes were assessed using the Kothari–Haber score, the absence of post-procedural imaging constitutes another limitation.

## Data Availability

The data that support the findings of this study are available on request from the corresponding author.

## References

[1] Siddiq MA Sood S Strachan D. Pharyngeal pouch (Zenker’s diverticulum). Postgrad Med J. 2001;77(910):506 511. (doi: 10.1136/pmj.77.910.506) 11470929 PMC1742115

[2] Dell’Anna G Fasulo E Fanizza J The endoscopic management of Zenker’s diverticulum: A comprehensive review. Diagnostics (Basel). 2024;14(19):2155. (doi: 10.3390/diagnostics14192155) PMC11475965 39410559

[3] Pang M Koop A Brahmbhatt B Bartel MJ Woodward TA. Comparison of flexible endoscopic cricopharyngeal myectomy and myotomy approaches for Zenker diverticulum repair. Gastrointest Endosc. 2019;89(4):880 886. (doi: 10.1016/j.gie.2018.09.043) 30342027

[4] Elkholy S El-Sherbiny M Delano-Alonso R Peroral endoscopic myotomy as treatment for Zenker’s diverticulum (Z-POEM): a multi-center international study. Esophagus. 2021;18(3):693 699. (doi: 10.1007/s10388-020-00809-7) 33387150

[5] Zhang LY Hernández Mondragón O Pioche M Zenker’s peroral endoscopic myotomy for management of large Zenker’s diverticulum. Endoscopy. 2023;55(6):501 507. (doi: 10.1055/a-2025-0715) 36827992

